# Enhanced Age-Related Resistance to Tomato Yellow Leaf Curl Virus in Tomato Is Associated With Higher Basal Resistance

**DOI:** 10.3389/fpls.2021.685382

**Published:** 2021-07-29

**Authors:** Jing-Ru Zhang, Shu-Sheng Liu, Li-Long Pan

**Affiliations:** Ministry of Agriculture Key Laboratory of Molecular Biology of Crop Pathogens and Insects, Institute of Insect Sciences, Zhejiang University, Hangzhou, China

**Keywords:** TYLCV, age-related resistance, salicylic acid, virus quantity, infection rate, plant biomass

## Abstract

Tomato yellow leaf curl virus (TYLCV) is one of the most notorious plant pathogens affecting the production of tomato worldwide. While the occurrence of age-related resistance (ARR) against TYLCV has been reported, the factors impacting its development remain unknown. We conducted a series of experiments with three tomato cultivars that vary in basal resistance to TYLCV to explore factors involved in the development of ARR. Our data indicate that ARR is more pronounced in tomato cultivars with higher basal resistance. Additionally, increased plant biomass in older plants does not contribute to ARR. Virus source plants with a younger age at initial inoculation facilitates virus acquisition by whiteflies. Finally, an analysis on plant hormones suggests that salicylic acid (SA) may play a major role in the development of ARR in tomato against TYLCV. These findings provide new insights into the developmental resistance in tomato against TYLCV as well as clues for the deployment of ARR in the management of diseases caused by TYLCV.

## Introduction

Plant viral diseases pose serious threats to the production of many crops worldwide (Jones, 2020). In recent decades, diseases caused by tomato yellow leaf curl virus (TYLCV) (family *Geminiviridae*, genus *Begomovirus*) have imposed substantial losses to tomato production (Navas-Castillo et al., 2011; Dhaliwal et al., 2019). TYLCV infection in tomato plants results in yellowing and reduced size of the apical leaves, curling of leaf margins and stunted plant growth (Cohen and Harpaz, 1964; Prasad et al., 2020). First discovered in Jordan Valley in the 1930s, TYLCV was biologically characterized and named in 1960s (Cohen and Harpaz, 1964). Whilst TYLCV was confined to a few countries in Middle East in the first few decades post its characterization, its global spread started in 1980s when the Israel and Mild strains emerged and its whitefly vectors invaded many regions worldwide (Lefeuvre et al., 2010). Subsequently, TYLCV has become one of the most important plant pathogens in tomato production in dozens of countries around the globe as well as a focus for research in plant virology (Lefeuvre et al., 2010; Mabvakure et al., 2016; Prasad et al., 2020). Under natural conditions, TYLCV is transmitted by whiteflies of the *Bemisia tabaci* complex in a persistent circulative manner (Fiallo-Olivé et al., 2020; Wang and Blanc, 2021).

To date, various measures have been adopted to control TYLCV, including chemical, cultural and physical strategies, and breeding resistant plants (Rojas et al., 2018). Chemical control of its whitefly vectors, whilst commonly used, has resulted in the development of pesticide resistance and deleterious effects on the environment and human health (Gilbertson et al., 2015; Basit, 2019). Successful implementation of some cultural and physical tactics such as the application of a whitefly host-free period, colored shading nets and polyester covers, have also been reported (Polston and Anderson, 1997; Ben-Yakir et al., 2012; Al-Shihi et al., 2016). Nevertheless, increasing resistance in tomato plants via breeding represents the most effective measure to combat TYLCV (Islam and Wu, 2017; Dhaliwal et al., 2019). In resistance breeding, the most widely used resistance genes are *Ty-1*, *Ty-2*, and *Ty-3* that originate from *Solanum chilense* and *S. habrochaites* (Dhaliwal et al., 2019). However, to date genetic resources against TYLCV have been found in only a few wild relatives of cultivated tomato and introgression of resistance into tomato cultivars may take years and end up unsuccessful (Dhaliwal et al., 2019). More importantly, resistance break-down has been found in southeast Spain where severe epidemics of TYLCV in tomato cultivars carrying the *Ty-1* gene were reported (Torre et al., 2018). Therefore, alternative measures to improve the resistance in tomato plants are needed.

Post entry of viruses into plants, the outcome of virus-plant interaction may vary from immune to severe disease progression depending on many intrinsic and environmental factors (Osterbaan and Fuchs, 2019). Environmental factors, such as temperature and water availability, may significantly impact the interactions between plants and plant pathogens such as viruses, leading to altered disease development (Velásquez et al., 2018). Intrinsically, plant resistance and infectivity of viruses represent the most important factors affecting virus-plant interactions. On the plant side, many factors such as plant cultivar and age, among others, have been shown to modulate resistance to viruses (Osterbaan and Fuchs, 2019). The increase of resistance with plant age, often referred to as age-related resistance (ARR), has been shown to substantially affect virus-plant interactions (Panter and Jones, 2002; Hu and Yang, 2019). For example, the susceptibility of pepper to tomato spotted wilt tospovirus and soybean to bean pod mottle virus decreased with the increase of plant age (Moriones et al., 1998; Beaudoin et al., 2009; Byamukama et al., 2015). Likewise, the expression of genetic resistance to TYLCV was shown to increase with plant age (Levy and Lapidot, 2008). However, our knowledge of ARR in tomato against TYLCV is limited. Many key questions remain, for example, does plant age impact virus quantity in plants and in turn virus transmission by whiteflies that acquire TYLCV from these plants? Moreover, the ecological and molecular mechanisms underlying ARR against TYLCV in tomato remain unknown.

In the present study, we explored the factors involved in ARR against TYLCV in tomato plants. First, we compared TYLCV resistance among three tomato cultivars. Second, we characterized the impact of plant age on TYLCV resistance in plants of these cultivars. Third, we examined the effects of plant biomass at inoculation on ARR. Fourth, we analyzed the effects of virus source plants with varying ages at initial inoculation on acquisition and transmission of TYLCV by whiteflies. Finally, we examined the roles of plant hormones in ARR. Our findings provide insights into how ARR develops in tomato against TYLCV and how it might be utilized to combat the diseases caused by TYLCV.

## Materials and Methods

### Plants and Insects

Three cultivars of tomato (*S. lycopersicum* Mill), namely Pufen7, Pufen5, and Hezuo903, and one cultivar of cotton (*Gossypium hirsutum* cv. Zhe-Mian 1793), were used. Tomato seeds were purchased from Shanghai Funong Seed Co., Ltd., and cotton seeds were provided by the Institute of Crop Sciences, Zhejiang University. All plants were grown in insect-proof greenhouses under natural lighting at 25 ± 3°C. Unless specified otherwise, all plants were watered with solutions containing 0.17 g/L macromineral water-soluble fertilizer (DeMei LvYuan, China). For insects, a culture of MEAM1 whiteflies of the *B. tabaci* complex (mt*COI* GenBank accession code: KM821540) was used. Whiteflies were reared on cotton plants in insect-proof cages in climate chambers at 26 ± 2°C, 60–80% relative humidity and 14/10 h light/dark cycles. In all experiments, newly emerged female whiteflies (0–3 days post emergence) were used. The purity of the whitefly culture was assessed every 3 months using PCR-restriction fragment length polymorphism and mt*COI* sequencing (Qin et al., 2013).

### PCR and Quantitative PCR Detection of TYLCV in Tomato Plants and Whitefly

For the detection of TYLCV in tomato plants, the first apical fully-expanded leaves were harvested and subjected to DNA extraction. For TYLCV detection in whiteflies, adults were collected as groups of 15 and then subjected to DNA extraction. DNA extraction was done using procedures described previously (Pan et al., 2017). PCR detection of TYLCV was performed with primers TYLCV-F (5′-ATCGAAGCCCTGATATCCCCCGTGG-3′) and TYLCV-R (5′-CAGAGCAGTTGATCATG-3′). Quantitative PCR (qPCR) analysis of TYLCV was performed using SYBR Premix Ex Taq II (Takara, Japan) and CFX96 Real-Time PCR Detection System (Bio-Rad, Unted States) with the primers TYLCV-RTF (5′-GAAGCGACCAGGCGATATAA-3′) and TYLCV-RTR (5′-GGAACATCAGGGCTTCGATA-3′) for TYLCV, and primers WF-Actin-F (5′-TCTTCCAGCCATCCT TCTTG-3′) and WF-Actin-R (5′-CGGTGATTTCCTT CTGCATT-3′) for whitefly *actin*, and Tom-Actin-F (5′-TGGAGGATCCATCCTTGCATCAC-3′) and Tom-Actin-R (5′-TCGCCCTTTGAAATCCACATCTGC-3′) for tomato *actin*.

### Standard Procedure of Agrobacteria-Mediated Virus Inoculation

An infectious clone of TYLCV isolate SH2 (GenBank accession code: AM282874.1), provided by Professor Xueping Zhou (Institute of Biotechnology, Zhejiang University), was used (Wu et al., 2006). To perform agro-inoculation, agrobacteria containing infectious clones of TYLCV were first cultured until OD600 reached 1.5–2.0, and then resuspension buffer (10 mM MgCl_2_, 10 mM MES, 200 μM Acetosyringone) was used to re-suspend the agrobacteria. Re-suspended agrobacteria were then incubated at room temperature for 1 h and 1 mL syringes were used to introduce the agrobacteria into leaves of tomato plants. In virus inoculation experiments using agrobacteria, 0.6 mL of agrobacteria solution was introduced into each seedling and the test plants were then cultivated for 28 days before being sampled for PCR and qPCR detection of TYLCV.

### Standard Procedure of Whitefly-Mediated Virus Inoculation

In virus inoculation experiments using viruliferous whiteflies, TYLCV-infected Hezuo903 plants that were agro-inoculated at two true-leaf stage and had grown to 7–8 true-leaf stage were used as the source of inoculum. Whiteflies were collected from the lab culture and released onto TYLCV-infected plants to feed for 48 h for virus acquisition. Viruliferous whiteflies were then collected and placed on leaves (enclosed with leaf-clip cages) of test plants for 48 h for virus transmission. The number of whiteflies per test plants was five. Leaf-clip cages were made as reported before (Ruan et al., 2007). Whitefly survival was recorded during virus transmission and the live whiteflies were collected at the end of transmission and subjected to TYLCV quantification. Immediately after whitefly removal, imidacloprid (20 mg/L) was sprayed to kill whitefly eggs and the plants were cultivated for a further 28 days before being sampled for PCR and qPCR detection of TYLCV.

### Comparison of TYLCV Resistance Among Three Tomato Cultivars

Tomato seedlings of the three cultivars were grown to two true-leaf stage. Virus inoculation was conducted using the standard procedure of agrobacteria-mediated virus inoculation (Variable I, Table 1). Measurement of plant height (stem length in centimeters from soil level to stem tip) was conducted 4 weeks post virus inoculation as described before (Murphy and Bowen, 2006). In addition, two experiments of virus inoculation by whiteflies were conducted: in experiment 1, the standard procedure of whitefly-mediated virus inoculation was used; in experiment 2, the durations of both virus acquisition and virus inoculation were increased to 96 h and the number of viruliferous whiteflies was increased to 10 per plant (Variable I, Table 1).

**TABLE 1:** Variables tested in experiments for comparing resistance to Tomato yellow leaf curl virus (TYLCV) among tomato plants of different cultivars, of the same cultivar with different ages and of the same age with different biomass as well as for examining the effects of age of inoculum virus-infected plants on virus acquisition and transmission by whiteflies.

**Variables**	**Method of virus inoculation**	**Duration of virus acquisition (h)**	**Virus inoculation via whitefly feeding**		**No. of treatments/No. of replicates**	**No. of plants per replicates**
			**Duration (h)**	**No. of whiteflies per plant**		
Variable I: TYLCV	Agrobacteria	n/a-	n/a	n/a	3/3	7–8
resistance among three	Whitefly: experiment 1	48	48	5	3/3	7–8
tomato cultivars	Whitefly: experiment 2	96	96	10	3/3	7–8
Variable II: TYLCV	Agrobacteria	n/a-	n/a	n/a	4/3	6–10
resistance among plants of four different ages	Whitefly	48	48	5	4/3	6–10
Variable III: TYLCV resistance among plants of the same age with three different biomass	Agrobacteria	n/a-	n/a	n/a	3/8–15	1
Variable IV: Effects of age of inoculum virus-infected plants on virus acquisition and transmission by whitefly	Whitefly	48	48	5	2/3	8–9

*n/a stands for not applicable.*

### Comparison of TYLCV Resistance Among Plants of Different Ages

Tomato seeds were sown on a weekly basis, and seedlings were transplanted when they had reached two true-leaf stage. For each of the three cultivars, four batches of seedlings with the age of 0, 7, 14, and 21 days post transplanting (DPT), respectively, were prepared. Typical images of seedlings at different ages are presented in Supplementary Figure 1. Comparison of TYLCV resistance among plants of the four age-batches were conducted using the standard procedures of agrobacteria-mediated and whitefly-mediated virus inoculation (Variable II, Table 1).

### Comparison of TYLCV Resistance Among Plants of the Same Chronological Age With Different Biomass

Pufen7 and Hezuo903 seedlings at 0 DPT were prepared. For each of the two cultivars, seedlings were divided into three groups. The three groups of seedlings were watered with water, a solution containing 0.17 g/L macromineral water-soluble fertilizer and a solution containing 0.34 g/L macromineral water-soluble fertilizer, respectively. At 21 DPT, virus inoculation was conducted for the three groups of plants using the standard procedure of agrobacteria-mediated inoculation (Variable III, Table 1).

### Comparison of Virus Acquisition and Transmission by Whiteflies When Plants Inoculated at Different Ages Were Used as the Source of Inoculum

Two age groups of Hezou903 plants at 0 and 21 DPT were prepared and then agro-inoculated. Four weeks later, whiteflies were collected from the lab culture and released onto these plants to feed for 48 h for virus acquisition. Some viruliferous whiteflies were then collected and subjected to analysis of virus quantity. Further, virus transmission capacity of the viruliferous whiteflies from plants of each of the two age groups were tested with Hezuo903 seedlings of 0 DPT using the standard procedure of whitefly-mediated virus inoculation (Variable IV, Table 1).

### Analysis of Salicylic Acid and Jasmonates Contents

Pufen7 and Hezuo903 plants at 0 and 21 DPT were prepared. For the analysis of salicylic acid (SA) and jasmonates (JA) contents, leaves from three seedlings were mixed and used as one sample. For each treatment (cultivar × plant age), four and nine-ten replicates were conducted for SA and JA, respectively. Samples were first powdered in liquid nitrogen and then 0.15 g of leaf powder was transferred into centrifuge tubes. Plant hormones were extracted using 1 mL of ethyl acetate containing 10 ng of D4-SA and D6-JA. All samples were vortexed and centrifuged and supernatants collected and concentrated using a vacuum concentrator. The dry residues were re-suspended in 110 μL of MeOH: H_2_O (50: 50, v/v). After mixing and centrifugation, 100 μL of the supernatants were collected. SA and JA were analyzed using an Agilent 6460 triple quadrupole mass spectrometer (Agilent Technologies, United States) equipped with an electrospray ionization (ESI) source that operated in the negative ion multiple-reaction monitoring (MRM) mode. Agilent Mass Hunter Workstation was used for data acquisition and processing.

### Quantitative Reverse Transcription PCR Analysis of SA Biosynthesis Genes

Seedlings of Pufen7 and Hezuo903 of different ages were prepared. For each treatment (cultivar × plant age), six replicates were conducted. Total RNAs were extracted with TRIzol and cDNA was synthesized using the PrimeScript RT reagent Kit with gDNA Eraser (Takara, Japan). qRT-PCR was performed using SYBR Premix Ex Taq II (Takara, Japan) and CFX96 Real-Time PCR Detection System (Bio-Rad, United States) with the primers ICS1-RTF (5′-GGCTTTAGCTGGAACACGG-3′) and ICS1-RTR (5′-CAATCTTCTTCTTATGCACTCCC-3′) for *ICS1*, and primers PAL-F (5′-CGTTATGCTCTCCGAACATC-3′) and PAL-R (5′-GAAGTTGCCACCATGTAAGG-3′) for *PAL*, and Tom-Actin-F (5′-TGGTCGGAATGGGACAGAAG-3′) and Tom-Actin-R (5′-CTCAGTCAGGAGAACAGGGT-3′) for tomato *actin*, and Tom-EF1α-F (5′-ATTGGAAATGGATATGCTCCA-3′) and Tom- EF1α-R (5′-TCCTTACCTGAACGCCTGTCA-3′) for tomato *EF1α*. Each gene was analyzed in two technical replicates.

### Statistical Analysis

For the analysis of virus quantity, real time data were calculated using 2 ^–△^^*Ct*^ as normalized to *actin*. For the analysis of gene expression level, real time data were calculated using 2^–△^^*Ct*^ as normalized to the geometric mean of two reference genes *actin* and elongation factor 1α (*EF1α*; Vandesompele et al., 2002). All percentage data were arcsine square root transformed for statistical analysis and back-transformed for presentation. For the comparison of virus transmission efficiency, virus quantity, plant hormone contents and gene expression level, one-way analysis of variance (ANOVA) along with Fisher’s least significant difference (LSD) was used when three or more treatments were conducted, and Student’s independent *t*-test was used when only two treatments were conducted. All data in this study were presented as the mean ± standard errors of mean (mean ± SEM). The differences between treatments were considered significant when *p* < 0.05. All statistical analysis was performed using SPSS 20.0 Statistics and EXCEL.

## Results

### Resistance to TYLCV in Three Tomato Cultivars

**FIGURE 1:**
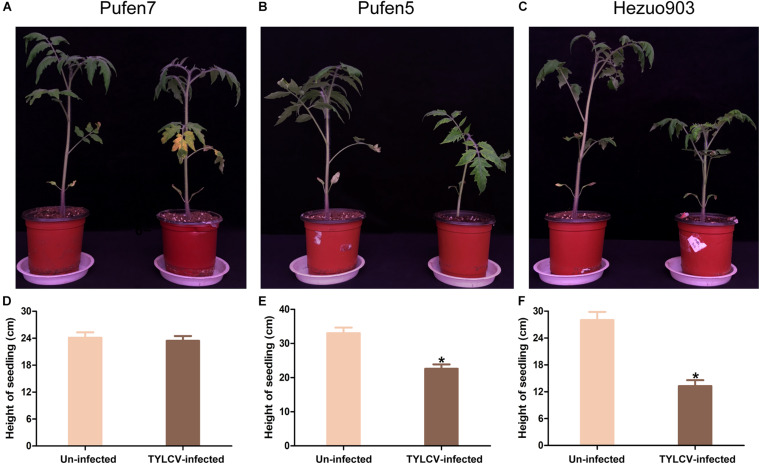
Typical images and height of un-infected and tomato yellow leaf curl virus (TYLCV)-infected tomato plants of different cultivars. **(A–C)** Image of Pufen7, Pufen5, and Hezuo903. **(D–F)** Height of un-infected and TYLCV-infected tomato plants. Photographing and measurement of plant height were conducted 4 weeks post virus inoculation. Values are means ± SEM in panels **(D–F)** (*n* = 8–15). *Above columns indicate significant differences (independent *t*-test, *p* < 0.05).

**FIGURE 2:**
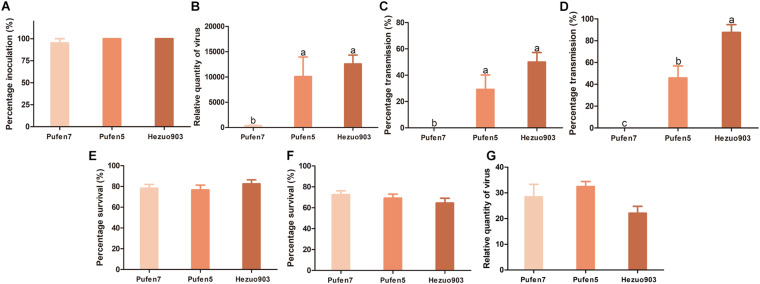
Resistance to tomato yellow leaf curl virus (TYLCV) in plants of tomato cultivars Pufan7, Pufen5, and Hezuo903. **(A)** Percentage of TYLCV-infected plants in agro-inoculated plants; **(B)** Relative quantity of virus in agro-inoculated plants; **(C)** Percentage of TYLCV-infected plants in whitefly-inoculated plants when the durations of both virus acquisition and transmission were 48 h and five whiteflies were used per test plant; **(D)** Percentage of TYLCV-infected plants in whitefly-inoculated plants when the durations of both virus acquisition and transmission were 96 h and ten whiteflies were used per test plant; **(E)** Whitefly survival on plants when five whiteflies were used per test plant; **(F)** Whitefly survival on plants when ten whiteflies were used per test plant; **(G)** Relative quantity of virus in whiteflies recovered from plants post virus transmission when 10 whiteflies were used per test plant. Values are means ± SEM (*n* = 3 for panels **(A,C,D)** and in each replicate 7–8 plants were used; *n* = 14 for panel **(B)**; *n* = 24 for panels **(E,F)**; *n* = 4 for panel **(G)**]. Different letters above the columns indicate significant differences (one-way ANOVA, *p* < 0.05).

Tomato yellow leaf curl virus infection did not induce noticeable symptom in Pufen7 plants (Figure 1A). In Pufen5 plants yellowing of several apical leaves was observed; in Hezou903 plants both yellowing and severe leaf curl were observed (Figures 1B,C). TYLCV infection significantly reduced the height of Pufen5 (by 32.1%) and Hezou903 (by 53.4%) plants, but did not affect the height of Pufen7 plants (Figures 1D–F). Some yellowing was observed in cotyledons and old leaves that were agro-inoculated in both un-infected and TYLCV-infected plants for each cultivar. Post agro-inoculation, 95.2% of Pufen7 plants and all the plants of Pufen5 and Hezou903 were infected by TYLCV (Figure 2A). The highest quantity of TYLCV was found in Hezou903, followed by Pufen5 and then Pufen7 (Figure 2B). Similarly, two whitefly transmission assays revealed that the highest TYLCV infection rate was always found in Hezou903, followed by Pufen5 and then Pufen7 (Figures 2C,D). Notably, no successful transmission was found for Pufen7 plants. During virus transmission, there was no significant difference in whitefly survival rate and TYLCV quantity in recovered whiteflies among plants of different cultivars (Figures 2E–G).

### Effects of Plant Age on TYLCV Resistance in Pufen7 Plants

**FIGURE 3:**
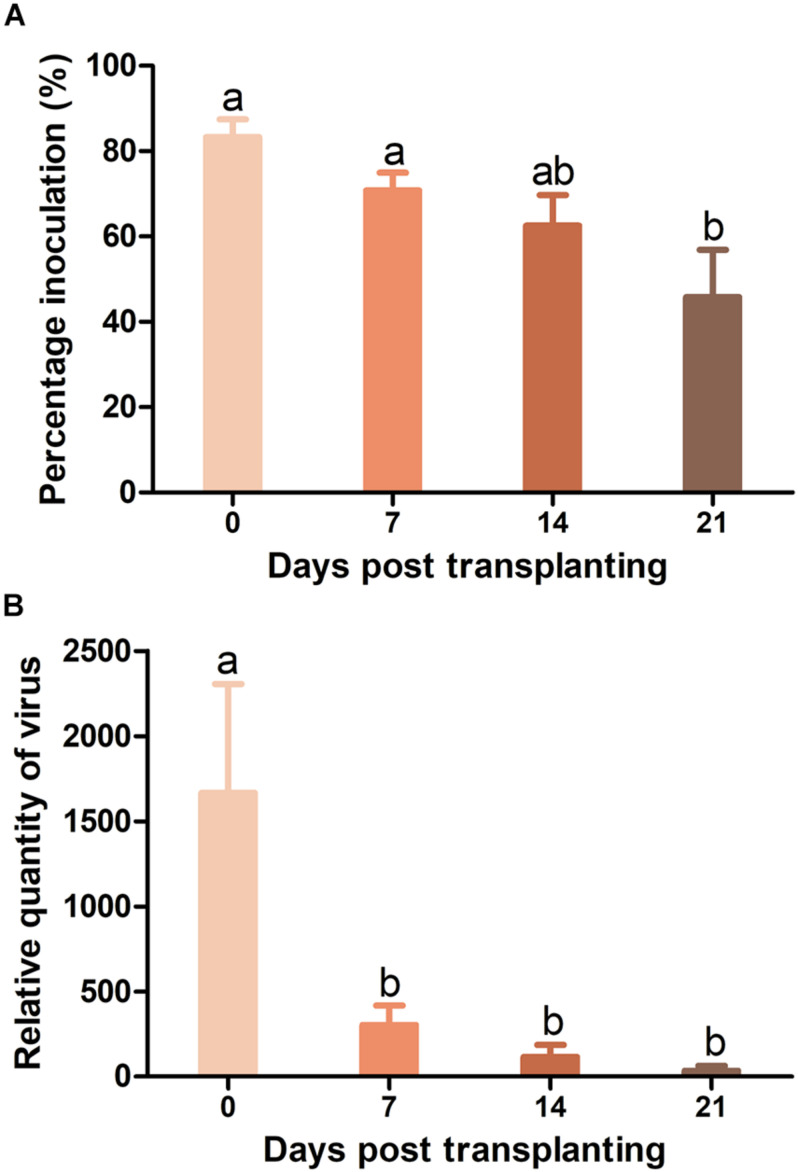
Resistance to tomato yellow leaf curl virus (TYLCV) in Pufen7 seedlings of 0, 7, 14, and 21 DPT. **(A)** Percentage of TYLCV-infected plants in agro-inoculated plants; **(B)** Relative quantity of virus. Values are means ± SEM (*n* = 3 and in each replicate 7–8 plants were used for panel **(A)**; *n* = 8 for panel **(B)**]. Different letters above the columns indicate significant differences (one-way ANOVA, *p* < 0.05).

As whiteflies were unable to transmit TYLCV to Pufen7 plants, TYLCV resistance in Pufen7 plants of different ages was assayed only by agro-inoculation and virus infection status was assessed 4 weeks post inoculation. TYLCV infection rate decreased sequentially from 83.3 to 45.8% with the increase of plants age (Figure 3A). An obvious reduction in TYLCV quantity was observed with the increase of plant age (Figure 3B).

### Effects of Plant Age on TYLCV Resistance in Pufen5 Plants

**FIGURE 4:**
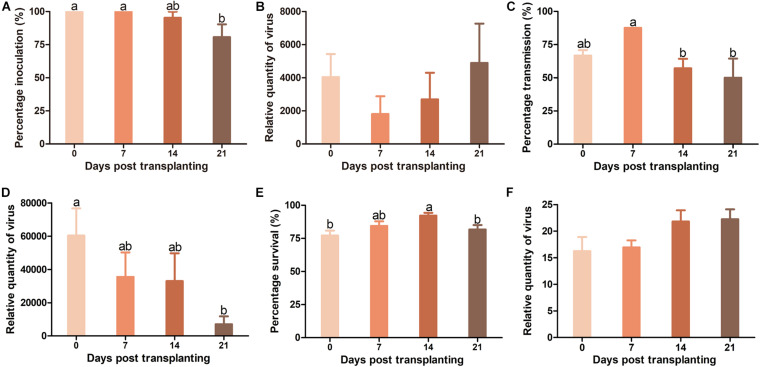
Resistance to tomato yellow leaf curl virus (TYLCV) in Pufen5 seedlings of 0, 7, 14, and 21 DPT. **(A)** Percentage of TYLCV-infected plants in agro-inoculated plants; **(B)** Relative quantity of virus in agro-inoculated plants; **(C)** Percentage of TYLCV-infected plants in whitefly-inoculated plants; **(D)** Relative quantity of virus in whitefly-inoculated plants; **(E)** Whitefly survival on tomato seedlings of different ages; **(F)** Relative quantity of virus in whiteflies recovered post virus transmission. Values are means ± SEM [*n* = 3 and in each replicate 7–8 plants were used for panels **(A,C)**; *n* = 7–8 for panels **(B,D)**; *n* = 21–24 for panel **(E)**; *n* = 4 for panel **(F)**]. Different letters above the columns indicate significant differences (one-way ANOVA, *p* < 0.05).

When TYLCV was agro-inoculated into plants, TYLCV infection rate in Pufen5 plants did not differ significantly between plants of 0 and 7 DPT. However, for plants of 7–21 DPT, a slight decrease of TYLCV infection rate from 100 to 80.7% was observed with the increase of plant age (Figure 4A). No significant difference was found for TYLCV quantity in plants of different ages post agro-inoculation (Figure 4B). When whitefly transmission was used, TYLCV infection rate decreased from 87.5% at 7 DPT to 50% at 21 DPT (Figure 4C). Sequential decreases of TYLCV quantity with the increase of plant age was observed (Figure 4D). For whiteflies, during virus transmission their survival rate on plants at 14 DPT (92.2%) was significantly higher than that on plants at 0 DPT (77.1%) and 21 DPT (81.7%) (Figure 4E). No significant difference in TYLCV quantity in whiteflies recovered from plants of different ages was found (Figure 4F).

### Effects of Plant Age on TYLCV Resistance in Hezuo903 Plant

**FIGURE 5:**
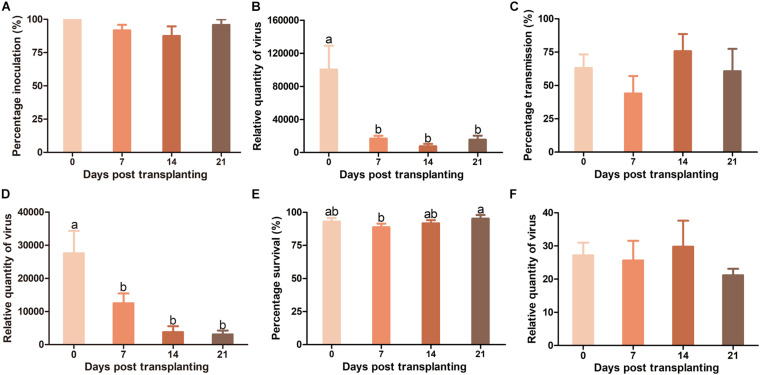
Resistance against tomato yellow leaf curl virus (TYLCV) in Hezuo903 seedlings of 0, 7, 14, and 21 DPT. **(A)** Percentage of TYLCV-infected plants in agro-inoculated plants; **(B)** Relative quantity of virus in agro-inoculated plants; **(C)** Percentage of TYLCV-infected plants in whitefly-inoculated plants; **(D)** Relative quantity of virus in whitefly-inoculated plants; **(E)** Whitefly survival on tomato seedlings; **(F)** Relative quantity of virus in whiteflies recovered post virus transmission. Values are means ± SEM [*n* = 3 and in each replicate 6–10 plants were used for panels **(A,C)**; *n* = 7–8 for panels **(B,D)**; *n* = 21–24 for E; *n* = 3 for panel **(F)**]. Different letters above the columns indicate significant differences (one-way ANOVA, *p* < 0.05).

There was no significant difference in TYLCV infection rate in Hezuo903 plants of different ages when TYLCV was agro-inoculated into plants (Figure 5A). However, TYLCV quantity in plants decreased significantly from 0 to 7 DPT and did not differ significantly among plants of 7, 14, and 21 DPT (Figure 5B). When whitefly transmission was used, there was no significant difference in TYLCV infection rate among plants of different ages (Figure 5C), but again TYLCV quantity decreased significantly with the increase of plant age (Figure 5D). For whiteflies, during virus transmission their survival rate on plants was similar except that survival rate on plants of 7 DPT was slightly lower than that on plants of 21 DPT (Figure 5E). There was no significant difference in TYLCV quantity in whiteflies recovered from plants of different ages (Figure 5F).

### Effects of Plant Biomass at Inoculation on Virus Quantity

**FIGURE 6:**
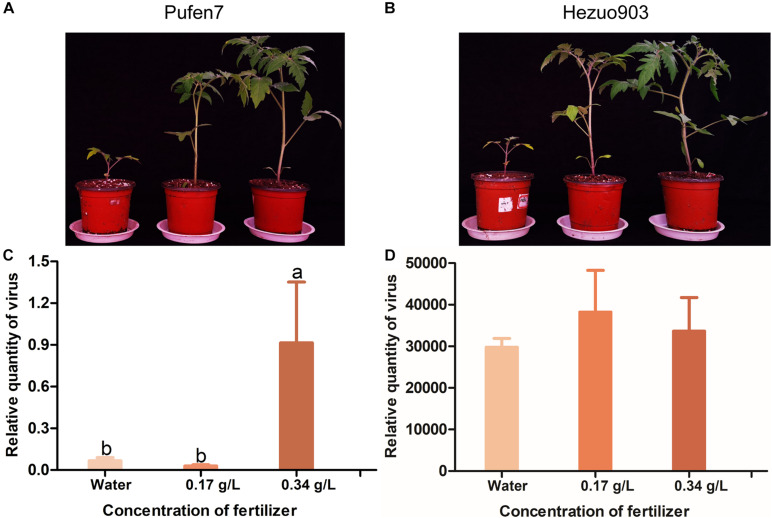
Picture of tomato seedlings that were treated with water or solutions containing different concentration of fertilizer for 21 days and relative quantity of virus in these plants post agro-inoculation. **(A)** Pufen7 seedlings; **(B)** Hezuo903 seedlings; **(C)** Relative quantity of virus in Pufen7 plants; **(D)** Relative quantity of virus in Hezuo903 plants. Values are means ± SEM in panels **(C,D)** [*n* = 15 for panel **(C)** and 8 for panel **(D)**]. Different letters above the columns in panels **(C,D)** indicate significant differences (one-way ANOVA, *p* < 0.05).

Plants differed in biomass (size) among the three fertilizer treatments for both Pufen7 (Figure 6A) and Hezuo903 (Figure 6B). In the data of relative quantity of virus of the three fertilizer treatments in Pufen7, we noted one outlier with exceptionally high virus quantity in each of the three treatments: 41.2, 18.9, and 23.1 for water, 0.17 g/L, and 0.34 g/L, respectively. After elimination of the outliers, Kolmogorov–Smirnov analysis showed that the remaining data followed a normal distribution and thus were analyzed using ANOVA. Following virus inoculation, virus quantity was significantly higher in plants watered with a solution containing 0.34 g/L macromineral water-soluble fertilizer than that in the other two treatments for Pufen7 (Figure 6C). Virus quantity did not differ significantly among plants of the three fertilizer treatments for Hezuo903 (Figure 6D).

### Effects of Virus Inoculum Plants Inoculated at Different Ages on Virus Acquisition and Transmission by Whiteflies

**FIGURE 7:**
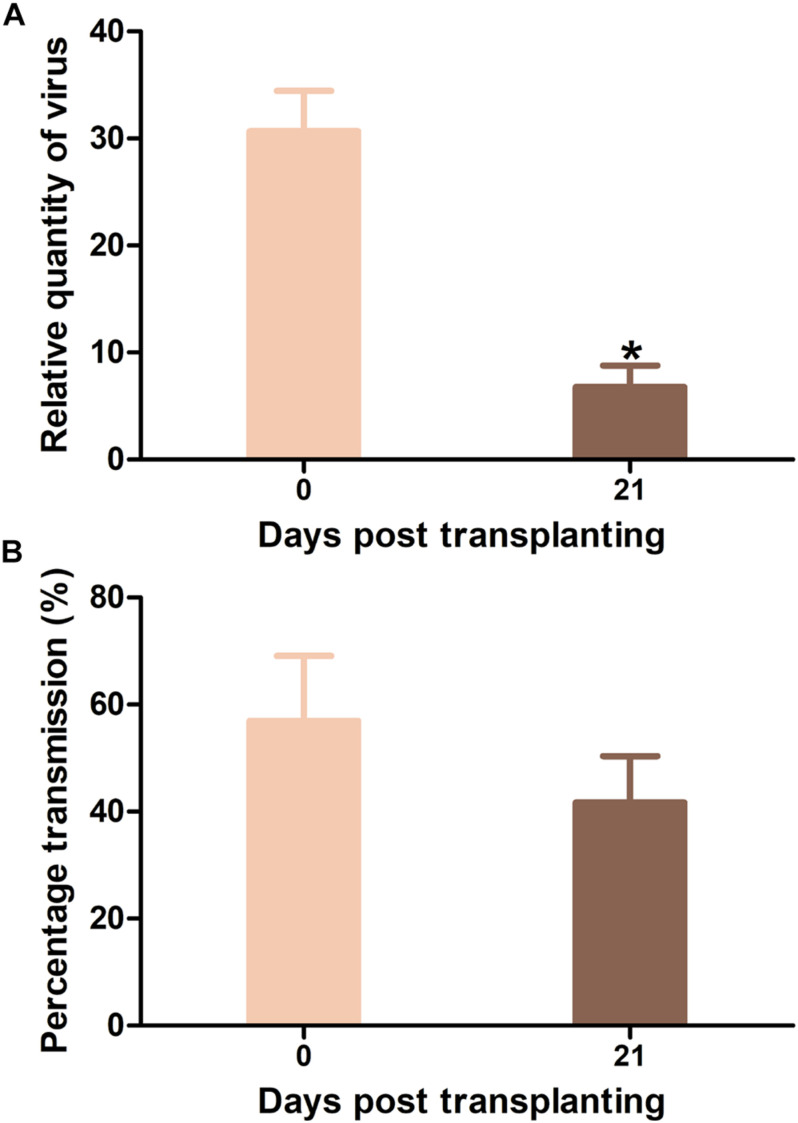
Tomato yellow leaf curl virus (TYLCV) quantity in whiteflies and virus transmission efficiency by whiteflies when Hezou903 plants inoculated at 0 and 21 DPT were used as the source of inoculum. **(A)** TYLCV quantity in whiteflies; **(B)** TYLCV transmission efficiency by whiteflies. Values are means ± SEM (*n* = 4 for panel **(A)**; *n* = 3 and each replicate contains 8–9 test plants for panel **(B)**. *Above columns indicate significant differences (independent *t*-test, *p* < 0.05).

Tomato yellow leaf curl virus-infected Hezuo903 plants that were agro-inoculated at 0 and 21 DPT were presented to whiteflies for virus acquisition. TYLCV quantity in whiteflies was significantly higher when the source of inoculum was agro-inoculated at 0 DPT than that at 21 DPT (Figure 7A). Virus transmission rate by whiteflies did not differ significantly between the two treatments (Figure 7B).

### Effects of Plant Age on the Contents of SA and JA

**FIGURE 8:**
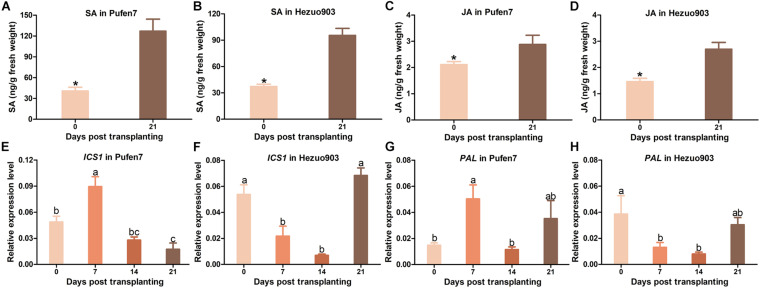
Contents of salicylic acid (SA) and jasmonates (JA) and relative expression of SA biosynthesis genes in Pufen7 and Hezuo903 seedlings. **(A)** Contents of SA in Pufen7 seedlings of 0 and 21 DPT; **(B)** Contents of SA in Hezuo903 seedlings; **(C)** Contents of JA in Pufen7 seedlings; **(D)** Contents of JA in Hezuo903 seedlings; **(E)** Relative expression level of *ICS1* in Pufen7 seedlings of 0, 7, 14, and 21 DPT; **(F)** Relative expression level of *ICS1* in Hezuo903 seedlings; **(G)** Relative expression level of *PAL* in Pufen7 seedlings; **(H)** Relative expression level of *PAL* in Hezuo903 seedlings. Values are means ± SEM [*n* = 4 for panels **(A,B)**; *n* = 9–10 for panels **(C,D)**; *n* = 6 for panels **(E–H)**]. * above columns in panels **(A–D)** and different letters in panels **(E–H)** indicate significant differences among plants of different ages [independent *t*-test, *p* < 0.05 for panels **(A–D)**; one-way ANOVA, *p* < 0.05 for panels **(E–H)**].

For both cultivars, the level of endogenous SA was significantly higher in seedlings of 21 DPT than that in seedlings of 0 DPT (Figures 8A,B). Similarly, endogenous JA level was significantly higher in seedlings of 21 DPT than that in seedlings of 0 DPT (Figures 8C,D). Expression of gene *ICS1* in Pufen7 increased significantly at the early stages and then decreased to levels lower than that at 0 DPT (Figure 8E). *ICS1* expression in Hezuo903 decreased significantly from 0 to 14 DPT but then increased to a level similar to that at 0 DPT (Figure 8F). For *PAL*, its expression in Pufen7 increased significantly from 0 to 7 DPT but decreased thereafter (Figure 8G). *PAL* expression in Hezuo903 decreased significantly from 0 to 14 DPT and then increased to levels similar to that at 0 DPT (Figure 8H).

## Discussion

In this study, we explored the influence of basal resistance to TYLCV in tomato on ARR using three cultivars that differ in TYLCV resistance. Our data indicate: (1) virus quantity in plants mostly decreased with the increase of plant age at the initial inoculation (Figures 3–5), (2) ARR appeared more evident in cultivars with higher basal resistance (Figures 3–5), (3) tomato plants with higher biomass at inoculation contained similar or higher quantity of TYLCV later in the plants (Figure 6), and (4) virus source plants with a younger age at initial inoculation facilitated virus acquisition by whiteflies (Figure 7). In addition, our analysis on plant hormones suggest that endogenous SA and JA level increased with plant age (Figure 8).

In the management of TYLCV, resistance breeding by introgression of genes from wild relatives of tomato into cultivated tomato has been considered as one of the most effective strategies in combating TYLCV (Islam and Wu, 2017; Dhaliwal et al., 2019). As the genetic resources of resistance from wild relatives of tomato are limited (Dhaliwal et al., 2019), employment of ARR may facilitate better utilization of them. Here we found that ARR is more pronounced in tomato cultivars with higher basal resistance. Hence, ARR can be used in combination with resistance from other sources such as *Ty-1*, *Ty-2*, and *Ty-3* to obtain higher overall resistance. Moreover, even in the susceptible cultivar Hezuo903, reduced TYLCV quantity was observed with increased plant age at initial inoculation. The reduced TYLCV quantity may in turn reduce the subsequent spread of the virus by whitefly vectors. Therefore, maintaining tomato seedlings free of TYLCV to an older age in well-protected nurseries before transplanting into open fields can be a cost-effective measure in disease management (Riley and Srinivasan, 2019).

Since plants of different ages differ significantly in biomass and resistance to TYLCV, we sought to explore the contribution of biomass in ARR. Intriguingly, tomato plants of the same chronological age with higher biomass at inoculation contained similar or higher quantity of TYLCV later in the plants, indicating that increased biomass in older plants did not contribute to ARR. As regards one outlier with exceptionally high virus quantity in each of the three fertilizer treatments for Pufen7 (Figure 6C), we suspect that the Pufen7 seeds used in the experiment, which we obtained from a commercial source, may contain a small proportion of seeds that were partially susceptible to TYLCV.

As plants grow, substantial changes of many important traits, such as physical barriers, chemical defense and innate immunity may occur and contribute to ARR (Hu and Yang, 2019). For example, physical barriers contributed to ARR in potato against potato virus Y by modulating the systemic movement of virions in the foliage (Chikh-Ali et al., 2020). In cucumber fruit ARR against *Phytophthora capsici* can be attributable to the increases in transcriptional level of R-genes and activity of resistance related transcription factors (Mansfeld et al., 2020). Additionally, SA or other components in the SA-signaling pathway may contribute to ARR in *Arabidopsis thaliana* against several pathogens (Carviel et al., 2010; Wilson et al., 2017). Here, we analyzed the contents of SA and JA, two major defense-related plant hormones (Pieterse et al., 2012; Pan et al., 2021; Ye et al., 2021); we found that both endogenous SA and JA increased with plant age. Since SA is well-known for conferring resistance against biotrophic pathogens such as viruses (Pieterse et al., 2012; Pan et al., 2021; Ye et al., 2021), we propose that SA may play a major role in ARR against TYLCV in tomato plants. However, whether JA contribute to ARR against TYLCV warrants further investigations. Further, gene expression analysis was conducted to determine whether increase in plant hormone level resulted from increase in expression level of plant hormone biosynthesis genes. So far, two SA biosynthesis pathways have been identified in plants, namely phenylalanine ammonia lyase (PAL)-mediated phenylalanine pathway and isochorismate synthase (ICS)-mediated isochorismate pathway (An and Mou, 2011; Chen et al., 2020). In the present study, we found that the relative expression level of *ICS1* and *PAL* did not increase appreciably when tomato plants became older, suggesting that the increased SA contents in older plants may be due to the accumulation of this hormone in plants during development.

SA is produced in a wide range of prokaryotic and eukaryotic organisms (An and Mou, 2011). In plants, SA plays important roles in regulating many biological processes including growth and immune response (Koo et al., 2020). In the SA-signaling pathway, SA binds to two classes of receptors NPR1 and NPR3/NPR4, thereby regulating the expression of downstream genes (Liu et al., 2020). So far, three main stages of plant-virus interactions, namely intercellular trafficking, long-distance movement and replication of viruses, have been found to be regulated by SA (Zhao and Li, 2021). In addition, SA may modulate plant-virus interactions indirectly by affecting other antiviral pathways such as RNA silencing, pathogen-associated molecular pattern-triggered and effector-triggered immunity (Campos et al., 2014; Yang et al., 2015; Palukaitis and Yoon, 2020). Here we found that SA may directly contribute to ARR in tomato against TYLCV. However, how SA modulates resistance in an age-dependent manner warrants further investigations.

Taken together, we have found that ARR against TYLCV in tomato plants is more evident in plants with higher basal resistance. We revealed that plant biomass does not contribute to ARR in tomato against TYLCV, but virus source plants with a younger age at initial inoculation facilitates virus acquisition by whiteflies. We also showed that SA may directly contribute to ARR. Our findings provide new knowledge of ARR in tomato against TYLCV as well as clues for the deployment of ARR in the management of diseases caused by TYLCV.

## Data Availability Statement

The original contributions presented in the study are included in the article/Supplementary Material, further inquiries can be directed to the corresponding author.

## Author Contributions

J-RZ, S-SL, and L-LP contributed to conception and design of the study. J-RZ and L-LP performed the experiments. J-RZ performed the statistical analysis. J-RZ wrote the first draft of the manuscript. S-SL and L-LP revised the manuscript. All authors contributed to manuscript revision, read, and approved the submitted version.

## Conflict of Interest

The authors declare that the research was conducted in the absence of any commercial or financial relationships that could be construed as a potential conflict of interest.

## Publisher’s Note

All claims expressed in this article are solely those of the authors and do not necessarily represent those of their affiliated organizations, or those of the publisher, the editors and the reviewers. Any product that may be evaluated in this article, or claim that may be made by its manufacturer, is not guaranteed or endorsed by the publisher.
