# Supplementary material for: Enhanced Age-Related Resistance to Tomato Yellow Leaf Curl Virus in Tomato Is Associated With Higher Basal Resistance
Source: Front Plant Sci. 2021 Jul 29;12:685382. doi: 10.3389/fpls.2021.685382 (PMC8358113; doi:10.3389/fpls.2021.685382)
Supplement: Supplementary file 1 [file Data_Sheet_1.docx]

Supplementary Material

## Supplementary Figures


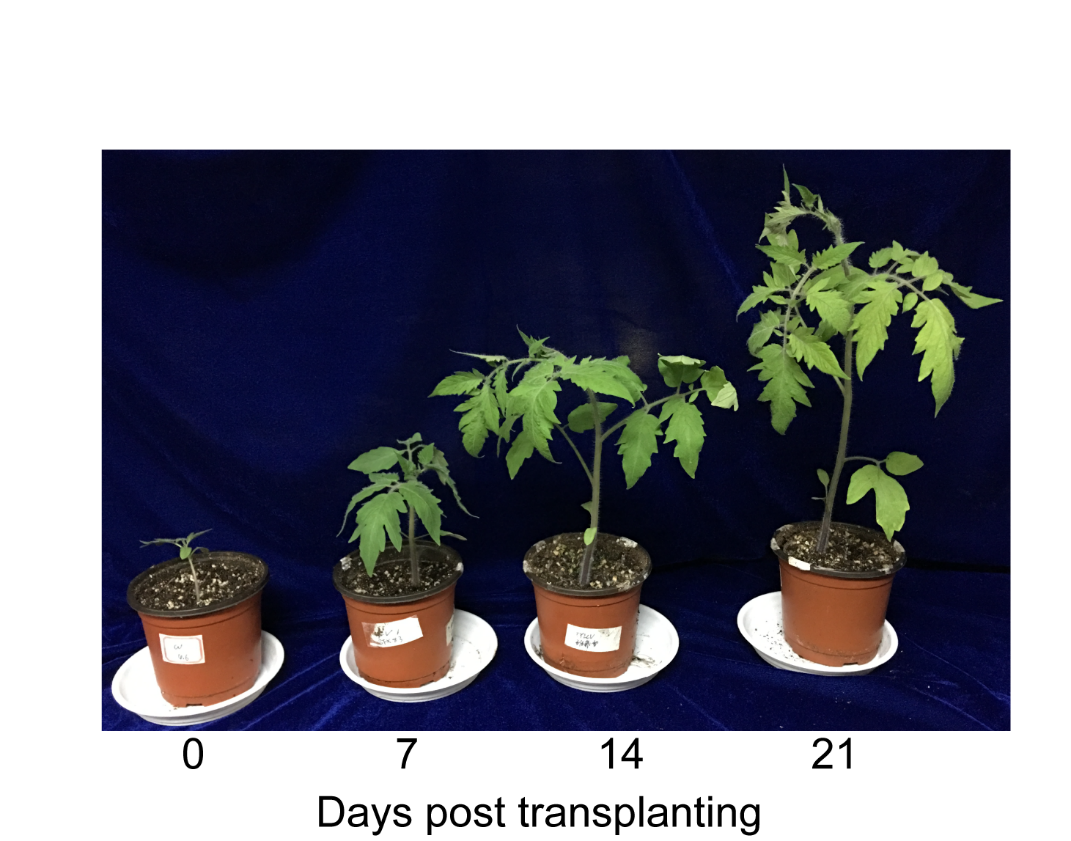


**Supplementary Figure 1.** Typical images of Pufen7 seedlings of 0, 7, 14 and 21 days post transplanting.
